# Modeling fear‐conditioned bradycardia in humans

**DOI:** 10.1111/psyp.12637

**Published:** 2016-03-07

**Authors:** Giuseppe Castegnetti, Athina Tzovara, Matthias Staib, Philipp C. Paulus, Nicolas Hofer, Dominik R. Bach

**Affiliations:** ^1^ Department of PsychiatryPsychotherapy, and Psychosomatics, University of ZurichZurichSwitzerland; ^2^ Neuroscience Centre Zurich, University of ZurichZurichSwitzerland; ^3^ Department of PsychologyDresden University of TechnologyDresdenGermany; ^4^ Wellcome Trust Centre for Neuroimaging, University College LondonLondonUK

**Keywords:** Psychophysiological model, Delay conditioning, Trace conditioning, Heart period, Skin conductance

## Abstract

Across species, cued fear conditioning is a common experimental paradigm to investigate aversive Pavlovian learning. While fear‐conditioned stimuli (CS+) elicit overt behavior in many mammals, this is not the case in humans. Typically, autonomic nervous system activity is used to quantify fear memory in humans, measured by skin conductance responses (SCR). Here, we investigate whether heart period responses (HPR) evoked by the CS, often observed in humans and small mammals, are suitable to complement SCR as an index of fear memory in humans. We analyze four datasets involving delay and trace conditioning, in which heart beats are identified via electrocardiogram or pulse oximetry, to show that fear‐conditioned heart rate deceleration (bradycardia) is elicited and robustly distinguishes CS+ from CS−. We then develop a psychophysiological model (PsPM) of fear‐conditioned HPR. This PsPM is inverted to yield estimates of autonomic input into the heart. We show that the sensitivity to distinguish CS+ and CS− (predictive validity) is higher for model‐based estimates than peak‐scoring analysis, and compare this with SCR. Our work provides a novel tool to investigate fear memory in humans that allows direct comparison between species.

Cued fear conditioning is a form of associative learning acquired by the contingent coupling of a neutral precursor (conditioned stimulus, CS+) with an aversive event (unconditioned stimulus, US). It is often seen as a laboratory model of phobia or posttraumatic stress disorder (VanElzakker, Dahlgren, Davis, Dubois, & Shin, 2014). This is why ongoing research programs seek to elucidate the neural microcircuits supporting this type of learning (Bach, Weiskopf, & Dolan, 2011; Ciocchi et al., 2010) and possibilities to prevent (Grillon, Cordova, Morgan, Charney, & Davis, 2004; Reist, Duffy, Fujimoto, & Cahill, 2001), or even erase (Kroes et al., 2014; Schiller et al., 2010) fear memory. Such investigations crucially rest on the ability to assess even subtle alterations in the strength of fear associations. Fear conditioning in many mammals, and particularly rodents, elicits overt behavioral responses to the CS+, such as freezing, which are easily quantified (LeDoux, Cicchetti, Xagoraris, & Romanski, 1990; Rogan, Staeubli, & LeDoux, 1997). This is not the case in humans, partly because very mild US are employed due to ethical considerations. Instead, typically the activity of the autonomic nervous system is assessed in human fear conditioning such as the sympathetically mediated skin conductance response (SCR, Boucsein, 2012; Collet, Vernet‐Maury, Delhomme, & Dittmar, 1997; Critchley, Elliott, Mathias, & Dolan, 2000). An alternative measure is fear potentiated startle (FPS, Brown, Kalish, & Farber, 1951; Hamm & Weike, 2005), which allows direct comparison between rodents (Falls, Carlson, Turner, & Willott, 1997; Walker, Ressler, Lu, & Davis, 2002) and humans (Hamm & Vaitl, 1996; Keil, Stolarova, Moratti, & Ray, 2007). However, FPS requires the presentation of aversive sounds during both CS+/CS− presentations and may thus interfere with the learning process, while autonomic measures can be obtained without interference. Hence, previous methodological research has sought to improve accuracy of fear memory quantification from SCR by model‐based methods (Bach, Daunizeau, Friston, & Dolan, 2010; Staib, Castegnetti, & Bach, 2015). Here, we investigated conditioned bradycardia as a complementary measure in humans. While both SCR (Boucsein, 2012) and cardiac responses (Berntson, Quigley, & Lozano, 2007; Bohlin & Kjellberg, 1978; Paulus, Castegnetti, & Bach, 2016) may be confounded by psychological arousal processes unrelated to fear memory, the combination of several psychophysiological techniques may provide more precise quantification.

In contrast to a long‐lasting, sympathetically mediated tachycardia observed during aversive contextual conditioning in rats (Nijsen et al., 1998; Roozendaal, Koolhaas, & Bohus, 1991), cue conditioning typically elicits short‐latency, parasympathetically mediated bradycardia in humans (Furedy & Poulos, 1976; Headrick & Graham, 1969; Klorman & Ryan, 1980) as well as rabbits (Gallagher, Kapp, McNall, & Pascoe, 1981; Gentile, Jarrell, Teich, McCabe, & Schneiderman, 1986) and rats (Supple & Leaton, 1990), thus providing comparability across species.

Stimulus‐evoked changes in heart rhythm are typically assessed in continuous data time series, created by interpolating instantaneous heart rate or heart period (Allen, Chambers, & Towers, 2007; Hodes, Cook, & Lang, 1985). In line with our previous work (Paulus et al., 2016), we use heart period here, because heart period and autonomic input are linearly related in vagal stimulation studies (Berntson et al., 2007). Our approach of creating continuous data renders the cardiac response amenable to model‐based methods (Paulus et al., 2016). These make prior assumptions on typical response shape and timing, embedded in psychophysiological models (PsPM, Bach & Friston, 2013). Such PsPMs can then be inverted in order to quantify autonomic input. This approach distinguishes between the response components of interest and what is treated as noise. This can improve signal‐to‐noise ratio and thereby reconstruction of the causes of observed data, as in the case of SCR (Bach, 2014; Bach & Friston, 2013), event‐related heart period response (HPR, Paulus et al., 2016) or fMRI (Friston, Jezzard, & Turner, 1994). In addition, one can define and separately interpret different components of the response and make inferences about the neural inputs responsible for these components. Finally, such a standardized approach could ameliorate the current heterogeneity in scoring the heart response found in the literature, thus ensuring a meaningful comparability between studies. Here, we develop and compare a set of methods based on general linear convolution models (GLM).

The goals of this study were threefold. First, we investigated to what extent HPR allow inference on associative fear memory in typical fear conditioning paradigms. Second, we sought to identify the best method to quantify fear from observed HPR. Finally, we compared the discriminative power provided by HPR and SCR measured during the same experiments.

Critically, the objective magnitude of fear memory (i.e., ground truth) is unknown to the experimenter. Here, we use a fear conditioning paradigm with many trials and with CS that are simple to learn, and assume that the CS+ will elicit a stronger autonomic response than the CS−. We can then assess how well a method recovers this difference between the two conditions; we term this predictive validity (Bach & Friston, 2013). Combined with Bayesian model comparison, this allows a statement on how much two methods differ in discriminating CS+/CS−, and hence in quantifying fear memory.

We built the methods on data from one delay conditioning experiment, and validated the results on three independent datasets: a delay conditioning experiment, a trace conditioning experiment, and a delay conditioning experiment with peripheral pulse oximetry rather than electrocardiography (ECG) for identification of heart beats. In the trace conditioning dataset, the CS/US interval was longer than in the other experiments. This allowed for the study of how to modify the model to account for small variations in the stimulus onset asynchrony (SOA). In particular, we expected the model to perform best when the anticipatory response is assumed to be time‐locked to the US, in agreement with previous findings (Damen & Brunia, 1987).

## Method

### Participants

We recruited four independent samples of healthy, nonmedicated individuals from the general population. All participants confirmed that they had no history of neurological, psychiatric, or systemic disorders, and all had normal or corrected‐to‐normal vision. We recorded data from 35 (23 females, age 18–31 years, mean ± *SD*, 23.4 ± 3.4 years), 20 (11 females, age 18–33 years, mean ± *SD*, 23.1 ± 3.7 years), 23 (10 females, age 20–32 years, mean ± *SD*, 23.8 ± 3.0 years), and 21 (8 females, age 19–34 years, mean ± *SD*, 25.7 ± 4.6 years) participants, respectively, in the four experiments. Because of technical malfunction or participants’ noncompliance with instructions, we excluded six subjects from Experiment 1, three subjects from Experiment 2, four subjects from Experiment 3, and four subjects from Experiment 4. All participants gave informed written consent before the beginning of the experiment. The study was conducted in accord with the Declaration of Helsinki and approved by the competent research ethics committee (Kantonale Ethikkommission Zürich).

### Experimental Procedure

#### Common settings

The unconditioned stimulus (US) was **a** train of electric square pulses delivered on participants' dominant forearm through a pin‐cathode/ring‐anode configuration with a constant current stimulator (Digitimer DS7A, Digitimer, Welwyn Garden City, UK). The current was set such that perceived shock intensity was around 90% of the pain threshold. We estimated the pain threshold during two phases. First, the intensity was increased from being unperceivable to a painful level. This was set as the upper threshold for the second phase, in which participants were asked to rate the perceived intensity of the delivered stimulus. These ratings were then interpolated to estimate the intensity that the subject would have rated as 90%. For all experiments, the screen had a diagonal of 20 inches, an aspect ratio of 16:9, and a resolution of 1,280 × 1,024 pixels at 50 Hz (P2014HT, Dell, Round Rock, TX). The duration of the intertrial interval was randomly determined to be 7, 9, or 11 s, and there were no habituation or extinction blocks. Reinforced trials were not analyzed. In all the experiments considered here, participants were not instructed about the contingency between CS and US and were asked to indicate stimulus identity by pressing one of two designated buttons on the keyboard. These designated buttons were counterbalanced across participants.

#### Experiment 1

Experiment 1 (dataset code: FR) implemented a delay fear conditioning paradigm with visual CS. For the US, we used 250 square electric pulses of 1‐ms duration and delivered at a frequency of 500 Hz, resulting in a total US duration of 0.5 s. Currents were between 1.0 and 6.7 mA (mean ± *SD*, 2.6 ± 1.28 mA). Participants were presented with 160 CS: 80 CS+, half of which coterminated with the US, and 80 CS− that predicted the absence of the US. The two CS types were two different colors (screen plain blue or red for CS+/‐) on a computer screen. The colors were counterbalanced across participants. The US was delivered 3.5 s after the CS onset; CS and US coterminated 0.5 s later.

#### Experiment 2

Experiment 2 (dataset code: SC4B) was a delay fear conditioning task with auditory CS. For the US, we used five square electric pulses with 0.2‐ms duration and delivered at a frequency of 10 Hz, resulting in a total US duration of 0.5 s. Currents were between 3.4 and 30.0 mA (mean ± *SD*, 10 ± 7.2 mA). A total of 192 trials was divided into eight blocks of 24 trials each. Of these, 96 were CS+, half of which coterminated with the US, and 96 were CS−. Two pairs of CS+ and CS−, either complex or simple, were delivered binaurally with headphones (HD518, Sennheiser, Wedemark‐Wennebostel, Germany) at about 68 dB. Complex stimuli were a sequence of four rising (400 to 800 Hz) or falling (800 to 400 Hz) sounds lasting 1 s each. Simple stimuli were tones with constant frequency (400 or 800 Hz) presented for 4 s. Within each block, only one pair of CS, either complex or simple, was presented. After 25% of the CS− and 50% of the nonreinforced CS+, a startle probe was delivered via headphones. These trials were not analyzed here. We confirmed with SCR that learning was not different for complex and simple CS and pooled them for the current analysis. To summarize, 96 trials were retained for the analysis, 72 of which were CS− and 24 nonreinforced CS+.

#### Experiment 3

This experiment (dataset code: TC) consisted of a trace fear conditioning task with the same CS, US, and settings as Experiment 1, with the exception that the CS/US onset asynchrony was 4 s instead of 3.5 s. Currents were between 1.0 and 7.0 mA (mean ± *SD*, 3.0 ± 1.3 mA). CS were presented for 3 s, after which a fixation cross appeared, followed 1 s later by the US in 50% of the CS+ trials.

#### Experiment 4

Experiment 4 (dataset code: VC1F) consisted of 16 blocks of 12 trials each, and was performed while participants underwent fMRI. Of the 16 blocks, eight consisted of explicitly instructed nonreinforced trials that are not analyzed here. The remaining eight blocks contained overall 96 trials, evenly divided into CS+, half of which coterminated with the US and CS−. The US were the same as in Experiment 2. Across participants, currents were set between 6 and 45 mA (mean ± *SD*, 17.2 ± 12.2 mA). Two pairs of visual CS of 4‐s duration were presented, either simple (during four blocks) or complex (during the other four blocks). Simple stimuli were two Gabor patches with different orientation (290° or 340°, counterbalanced across participants), while complex stimuli consisted of simple stimuli overlaid with an additional Gabor patch oriented at 230°.

#### Psychophysiological recording

In Experiments 1–3, ECG was recorded via four 45‐mm, pregelled Ag/AgCl adhesive electrodes attached to the four limbs. The experimenter visually identified the lead (I, II, III) or the augmented lead (aVR, aVL, aVF) configuration that displayed the highest R spike, and only recorded this configuration. Data were preamplified and 50 Hz notch‐filtered with a Coulbourn isolated five**‐**lead amplifier (LabLinc V75‐11, Coulbourn Instruments, Whitehall, PA), digitized at 1000 Hz using a Dataq card (DI‐149, Dataq Inc., Akron, OH) and recorded with Windaq (Dataq Inc.) software. In Experiment 4, the cardiac activity was detected at 500 Hz via a peripheral pulse sensor (PPS, SpO2 adult grip, Invivo, Gainesville, FL) placed around the nondominant index finger and connected to a wireless peripheral pulse unit via optic fib**e**r. This was transmitted to a wireless triggering unit and then to the MRI console for recording. We also recorded the SCR from the thenar/hypothenar of the nondominant hand using two 8‐mm disk Ag/AgCl cup electrodes (EL258, Biopac Systems Inc., Goleta, CA) and 0.5% NaCl gel (GEL101, Biopac; Hygge & Hugdahl, 1985). SC signal was measured with an SCR coupler/amplifier (V71‐23, Coulbourn Instruments) and digitized at 200 Hz. In Experiment 4, SCR was recorded with a Biopac MP150 data acquisition system coupled to a GSR‐100C signal amplifier (Biopac) at 1000 Hz sampling frequency.

### Data Preprocessing

Data processing and analysis was performed with MATLAB (Version R2013b, MathWorks Inc., Natick, MA). A modified offline implementation (Paulus et al., 2016) of the Pan & Tompkins (1985) real‐time QRS detection algorithm was used to identify QRS complexes from the ECG recording. Interbeat intervals (IBIs) deviating by more than two standard deviations from the single subject average were visually checked by a trained expert (GC) and corrected. To extract the heart beats from the PPS time series, we used a custom template‐matching algorithm. In particular, we obtained the template from the average waveform of the peaks that satisfied two conditions: first, a prominence higher than one‐third of the signal amplitude; second, a time distance from neighboring peaks higher than 0.3 s (corresponding to a heart rate of 200 beats per minute). The time points at which the correlation between the template and the PPS trace peaked were then assumed to correspond to heart beats. This assumption neglects the phase lag between the peripheral measure and the actual heart beat. However, the model developed with Dataset 1 (with ECG measures) well generalized to PPS data, suggesting this phase lag to be negligible for model‐based analysis, in line with previous findings on SCR (Bach, Flandin, Friston, & Dolan, 2010). Both with the ECG and the PPS, the IBI was assigned to its following heart beat, and the time series was interpolated linearly at 10 Hz to create equidistant data points. Interpolated heart period time series were band‐pass filtered with a bidirectional Butterworth filter: Unless otherwise specified, the low‐pass and the high‐pass cutoffs were 2 and 0.01 Hz, respectively. Single‐trial responses were analyzed in a time window of 11 s starting from the CS onset, corresponding to the minimum time interval between subsequent CS onsets. Single‐trial responses were baseline corrected by subtracting the heart period average during the 5 s before the CS onset, in line with previous research (Pollatos, Herbert, Matthias, & Schandry, 2007). This baseline window reconciles the need to average out respiratory arrhythmia and to minimize the effect of the previous trial. SCR data were preprocessed with a Butterworth band‐pass filter with 0.0159 Hz and 5 Hz cutoff, respectively. For statistical analysis of the SCR, we used the default dynamical causal model (DCM) method as implemented in PsPM 3.0 (http://pspm.sourceforge.net) (Bach, Daunizeau et al., 2010; Staib et al., 2015).

### Model Specification

We modeled the HPR as a linear time invariant system (LTI). This is a system with two characteristic properties: First, the output does not explicitly depend on time (time invariance), and second, the response to several inputs is the sum of the responses to the individual inputs (linearity). In most real systems, including the heart, these criteria are only approximately met. In particular, the assumption of linearity implies pure summation of overlapping inputs, which may be unrealistic for the cardiac oscillator (Zebrowski et al., 2007). However, we assume that, with our choice of the intertrial interval, this approximation is accurate enough for the LTI formalism to be applicable. Thus, if an input x(t) produces the output  y(t), then the input x(t+δ), with δ∈R, elicits y(t+δ). An LTI system is fully specified by its response function (RF)  h(t). Recalling the operation of convolution between the functions *x* and *h* to be defined as

$$\left(x*h\right)\left(t\right)=\int_{0}^{\infty }x\left(\tau \right)h\left(t-\tau \right)d\tau ,$$

then one can obtain the response of a LTI system to any input by convolving it with the RF, that is,

$$y\left(t\right)=h\left(t\right)*x\left(t\right).$$

Here, we assume a short input at CS onset. The RF *h* then summarizes all neural and cardiac processes that finally lead to the heart period response.

There are at least two principled ways to construct a RF for an LTI. First, the RF can be formalized from known biophysical relationships between input and output. This is useful when dealing with biological systems whose mechanisms are largely known to the modeler (Friston, Mechelli, Turner, & Price, 2000). Secondly, if one or more internal states of the system are unknown and not accessible by experiment, a phenomenological RF must be inferred from the data. To this end, a set of known inputs is delivered to the system and the output measured. We capitalize on this second approach, which has also led to the successful development of a model for SCR (Bach, Flandin et al., 2010) and event‐related HPR (Paulus et al. 2016).

#### General linear models (GLM)

Once the shape of the RF is defined, the goal is to estimate the system's input to best explain data. If we assume the input to be of constant shape, we can harness GLM to estimate its amplitude. The assumption of a constant input shape is a simplification to increase robustness of the estimates. Specifically, we note that heart period variability due to the respiratory sinus arrhythmia (RSA) is typically larger than that induced by fear bradycardia. This means that trial‐by‐trial estimates of input into the cardiac system are corrupted by a signal that, at a first approximation, is not related with the emotional content of the CS. Assuming a constant shape allows averaging across many trials, thus removing RSA.

If *Y* is a set of *k* observations and *X* is the design matrix, the GLM can be written as

Y=Xβ+ɛ,

where ɛ is normally distributed noise. In our case, the columns of the design matrix *X* contain the time series (i.e., impulse functions at the onsets) of the different kinds of inputs representing the experimental design, convolved with the components of the RF. In other words, we define each column of *X* as a series of impulses located at the onset of the respective input type, convolved with the RF. Finally, the amplitude parameters β are estimated as the vector of coefficients for which the columns of *X* must be multiplied to obtain the best fit to the experimental time series (i.e., amplitudes of each component of RF). To infer β, the Moore‐Penrose pseudoinverse $X^{+}$ was calculated with a maximum likelihood inversion method as implemented in the MATLAB function pinv (Bach, Flandin, Friston, & Dolan, 2009).

#### Model construction

We sought to develop a data‐driven response function for discriminating between HPR to CS+ and CS− from the first dataset. To this end, we built the RF from the difference between the grand means of the responses to the two different stimuli. The shape of the response was determined by visually identifying, among different function classes, the function that qualitatively best resembled the difference between grand means. This suggested a gamma distribution to be a good candidate. Hence, we fitted it by finding the values of the shape parameter *k*, the scale parameter *ϴ*, the time onset *x_0_*, and the amplitude *A* that minimized the residual sum of squares (RSS) from the gamma distribution

$$y=\frac{A}{\theta^{k}\Gamma (k)}{\left(x-x_{0}\right)}^{k-1}e^{-\frac{x-x_{0}}{\theta }}.$$

**Figure 1:**
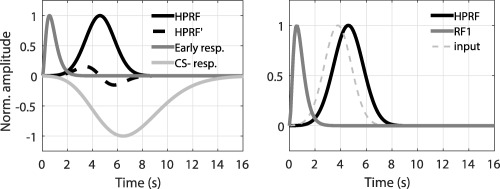
Left: Components of the response function. The four components that we combined to build the RFs (canonical response and its time derivative, early response, and response resulting by fitting the CS‐ only) are shown. The amplitudes are normalized for the sake of illustration. Right: Reconstruction of the autonomic input that convolved with the earliest HPR component found in our previous study (Paulus et al., 2016) most likely recovers the conditioned bradycardia response observed in the present work. The estimated autonomic input peaks at 3.5–4 s, that is, during anticipated presentation of the US.

The amplitude *A* is later left as a free parameter in the GLM implementation. We term this the canonical heart period response function (HPRF) and formalize it as model *G1* (Figure 1). To allow for subject‐specific variations in peak latency, we included its time derivative (HPRF’ in Figure 1) as a second component in models *G2* and *G3*, analogous to previous approaches (Bach et al., 2009; Friston et al., 1998). Finally, we observed an early response to both CS which might be interpreted as resulting from stimulus processing (Barry, 1982), and was formalized in a previous study (Paulus et al., 2016). We added this as a third component to *G3* (Figure 1). The previous models capitalize mainly on the difference between CS+ and CS−; however, a nonzero response to the CS− is also observed. An additional model *S1* combined this (Figure 1) with the canonical response reflecting the CS+/CS− differences. The GLMs *G1*, *G2*, and *G3* contained the canonical response as the first component, while *S1* included it as the second component. To estimate autonomic input from these different models, we reconstructed the estimated HPR from the entire basis set and calculated the signed maximal variation from baseline of this reconstructed response between 2 and 11 s after CS onset, in line with previous SCR work (Bach, Friston, & Dolan, 2013).

To allow broad application of our model in future investigations, we sought to base the HPRF on a larger dataset. Hence, we combined data from Experiments 1 and 2 to update the HPRF. We then studied its performance on the third dataset, thus checking the consistency of the method and setting the state of art for further developments.

Finally, we note that the assumption of constant, instantaneous input at CS onset is a mathematical simplification, not a biophysical reality, in particular because the CS extends in time. To assess the biophysical plausibility of the HPRF, we related it to previously reported HPR to brief stimuli for which the assumption of an almost instantaneous autonomic input is better justified. We specified the autonomic input into the previously identified LTI system that would produce the HPRF observed in the current study. We specified the input as a Gaussian function for which we estimated parameters by ordinary least square minimization (Figure 1).

#### Model comparison and validation

To find the model that best distinguishes CS+ and CS−, we used Bayesian model comparison between the models described above and model‐free peak‐scoring methods. As model‐free methods, we scored the HPR (a) by the amplitude of the maximum positive peak in a time window between 2 and 11 s after the CS onset (Furedy & Poulos, 1976), (b) by the signed amplitude of their maximal variation from baseline in this window (Geer, 1964), and (c) by the average HPR within a window of 2–8 s (Hermans, Henckens, Roelofs, & Fernández, 2013). The interval for method (c) is shorter because the average is a function of the entire data in the window and thus more susceptible to noise that occurs after the true response ends. To maximize the performance of this method, we optimized the time window on the first dataset. For all methods, we excluded the first 2 s because the HP is reported to vary in a nonspecific way in such window (Hermans et al., 2013). To quantify predictive validity, we calculated evidence for a model in which CS+ and CS− estimates are drawn from distributions with different means, rather than the same mean (analogous to a paired *t* test). We did this by computing a regression model in which the vector of event types is the dependent variable, and the vector containing the estimated response amplitudes is a regressor, complemented by regressors for subject‐specific intercepts (equivalent to a repeated measures analysis of variance, ANOVA). We then converted the RSS from this regression model into a negative log likelihood (NLL, Burnham & Anderson, 2004)

$$NLL=n\log\left(\frac{1}{n}RSS\right),$$

where *n* is the number of observations. A smaller NLL indicates a higher model evidence. We did not account for the number of parameters in the predictive model because it was the same for all approaches. An absolute NLL difference of more than 3 is often regarded as decisive, by analogy to a classic *p* value. If a classic test statistic falls into the rejection region, the probability of the data given the null hypothesis is *p* < .05. For an absolute NLL difference higher than 3, the probability of the null hypothesis given the data is *p* < $e^{-3}≃$ .05 (Penny, Stephan, Mechelli, & Friston, 2004; Raftery, 1995). Together with the NLL, we also report *t* and *p* values from an equivalent paired *t* test for illustration. Note that this slightly deviates from a previous approach where the condition (e.g., CS type) predicts the data (Bach et al., 2009; Green, Kragel, Fecteau, & LaBar, 2014). In both approaches, *t* or *F* values monotonically relate to predictive validity. However, in the previous approach, model evidence cannot be compared between the models. This is because model evidence scales with the dependent variable, which is then different between the models.

To investigate the effects of the CS−US SOA on the HPR, we designed Experiment 3 with a different SOA (4 s, instead of 3.5 s as in Experiment 1, 2, and 4). We tested two parsimonious models: (1) the RF is unchanged in shape and time‐locked to the CS, and (2) the RF is unchanged in shape and time‐locked to the US. We then compared the predictive validity of these two models to identify the most likely transformation that the RF undergoes as a function of the SOA.

Moreover, we sought to empirically rule out any bias of the model toward higher scorings of CS+ with respect to CS−. To do this, we randomly permuted the trial indices. This created two sets of trials between which the true autonomic inputs did not systematically differ. We then analyzed such mislabeled responses with the most discriminative method. To exclude any possible effect of the particular permutation, we performed the statistical analysis of the scores averaged over 1,000 different permutations.

#### Filter optimization

The settings of the Butterworth filter applied to the data might have an impact on the model performances. If the true RF was known, one could use the matched filter theorem to minimize the signal‐to‐noise ratio. However, as the RF is unknown, and may vary between subjects, we used Bayesian model comparison to optimize filter parameters in line with previous approaches (Bach et al., 2013; Staib et al., 2015). We varied the high‐pass cutoff between 0.01 and 0.1 Hz, and the low‐pass cutoff between 0.25, and 1 Hz, recomputed the RF, and re‐estimated CS+ and CS− responses. Similarly to the model selection, we optimized filter settings on the first dataset.

## Results

First, we used an established measure to confirm that participants successfully learned the CS/US association. SCR to CS+ were significantly larger than to the CS− (Experiment 1: *t*(28) = 3.23, *p* < .01; Experiment 2: *t*(16) = 4.67, *p* < .001; Experiment 3: *t*(18) = 3.58, *p* < .01; Experiment 4: *t*(16) = 2.90, *p* < .05) demonstrating the successful learning of the association between CS and US.

### Heart Period Responses

**Figure 2:**
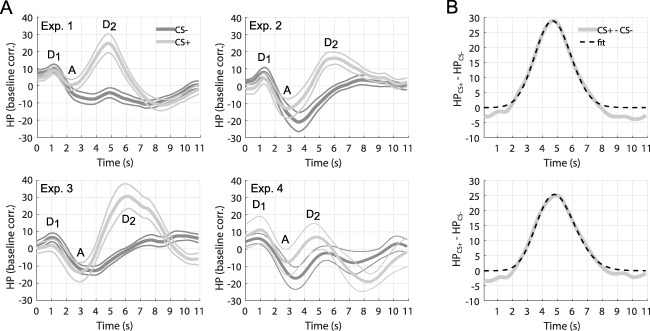
Heart period response. A: Response to CS+ and CS‐, averaged across participants and trials, ± *SEM* (thin lines), obtained with default filter settings (LP = 2 Hz, HP = 0.01 Hz) from the four datasets. Responses to CS typically begin with an initial deceleration (D_1_), followed by an acceleration (A), and a second deceleration (D_2_). B: Difference between the mean response to the CS+ and to the CS‐ (solid gray) and the best fitting gamma distribution (dashed black), obtained from Experiment 1 (upper) and after merging data from Experiment 1 and 2 (lower).

The grand means of the HPR to CS+ and CS− in the four experiments are depicted in Figure 2A. Importantly, responses in all experiments show the appearance of the three well‐known components of the cardiac response in two‐stimulus paradigms: an early deceleration (D_1_), followed by an acceleration (A), and a further late deceleration (D_2_; Bohlin & Kjellberg, 1978). Figure 2A shows that the HPR to CS+ is higher than the response to CS−, between 2 and 8 s after the CS onset. Since the two responses begin to differ after about 2 s (i.e., 1.5 s before the US onset), the bradycardia appears to be due to the CS presentation rather than the US omission. Three model‐free methods showed a highly significant CS+/CS− difference of the HPR (Table 1). Together, these results demonstrate the elicitation of anticipatory fear bradycardia as a response to fear‐conditioned stimuli.

**Table 1:** Model Comparison

#	Model description	NLL	*t*(28)	*p*
*P1*	Maximum variation from baseline	−97.0	3.0	5.0·10^−3^
*P2*	Peak scoring	−112.1	4.5	1.0·10^−4^
*P3*	Average in the 2–8 s window	−119.4	5.2	1.7·10^−5^
*G1*	HPRF	−115.7	4.8	4.3·10^−5^
*G2*	HPRF + HPRF’	−124.5	5.7	4.7·10^−6^
*G3*	HPRF + HPRF’ + early response	−124.1	5.7	4.7·10^−6^
*S1*	HPRF + baseline response	−115.2	4.8	4.9·10^−5^

*Note*. Listed are the values of the negative log likelihood (NLL) together with the *t* and the *p* values obtained by contrasting all the model‐free and the model‐based evaluations of the HPR to CS+ and CS‐. Absolute differences in NLL higher than 3 indicate significant differences in model evidence. The models *G2* and *G3* outperform the other model‐based and all the model‐free methods.

### Response Function

The difference between the responses to CS+ and CS− in Experiment 1 is depicted in Figure 2B. A heuristic function search suggested that a gamma distribution could best capture its shape. The fitted parameters of the gamma distribution, describing the shape of the canonical HPRF, are listed in Table 2. The amplitude of the canonical response and of additional model components is left as free parameters in the GLM inversion. From this canonical HPRF, we built different models, including only the HPRF (*G1*), adding its derivative (*G2*), adding derivative and an early response (*G3*), or the HPRF with the CS− response (*S1*).

**Table 2:** Parameters of the Gamma Distributions Modeling the HPRF

	*k*	*ϴ*	*x_0_*
Dataset 1	2.56·10^5^	2.26·10^−3^	−574
Dataset 1 optimized	1.37·10^3^	3.11·10^−2^	−38.0
Dataset 1 + 2	43.2	0.196	−3.47
Dataset 1 + 2 optimized	48.5	0.182	−3.86

*Note*. The parameters *k* (shape parameter), ϴ (scale parameter) and *x*_0_ (time onset) of the gamma distribution resulting from the fit to the difference between response to CS+ and CS‐. These parameters were obtained after averaging the responses over the first dataset only or a combination of the first and the second dataset, before and after filter optimization.

### Model Comparison

For each model (*G1–3* and *S1* in Table 1), we computed the predictive validity of their amplitude parameters (Table 1), that is, their ability to discriminate between CS+ and CS−, and compared it with model‐free methods (*P1–3*), based on the first dataset. As a first result, all model‐based methods discriminated CS+ and CS−. Secondly, the two best model‐based methods (*G2/G3* in Table 1) are the ones with the derivative of the gamma distribution as second component, with or without the early response. Finally, the best model‐based methods were more predictive than the best model‐free method (*P3*). There was no difference between the two best models such that we defined the simpler model *G2* as winning model.

### Filter Settings

**Figure 3:**
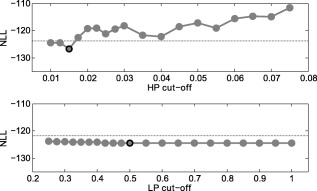
Comparison between filter settings. The figure shows predictive validity as negative log‐likelihood (smaller is better) for model *G2* in dependence on the high‐pass (HP) cutoff (top) and on the low‐pass (LP) cutoff (bottom). The high‐pass cutoff has a strong effect on the predictive validity, returning the best performance between 0.01 and 0.015 Hz. Conversely, the low‐pass cutoff does not significantly affect the predictive validity. The dashed lines represent the significance thresholds with respect to the selected winning cutoffs (circled dots).

We next searched for the filter settings that maximize predictive validity of the winning model. The results are shown in Figure 3. The high‐pass cutoff that returned the best predictive validity was 0.015 Hz. Cutoff frequencies between 0.01–0.015 Hz were not significantly worse. Instead, the low‐pass filter did not significantly affect the performance of the model. We therefore chose 0.015 Hz as high‐pass cutoff and an arbitrary 0.5 Hz cutoff for the low‐pass filter as it was a frequency in the middle range of the low‐pass cutoffs analyzed. The parameters obtained after fitting the difference between CS+ and CS− with these filters are listed in Table 2. With these settings, predictive validity was slightly but not significantly improved compared to the original filter settings (NLL = −126.7 for new filter settings, NLL = −124.6 for original filter settings).

### Model Validation

**Figure 4:**
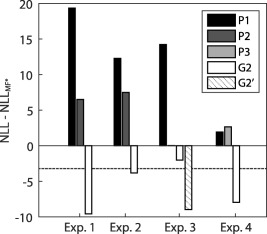
Model comparison. Bars represent predictive validity as negative log‐likelihood (NLL, smaller is better) for three model‐free methods (*P1–P3*) and the winning model *G2* with the optimal filter settings (HP cutoff: 0.015 Hz, LP cutoff: 0.5 Hz), including the canonical HPRF and its time derivative. For each experiment, the NLL was normalized to the best model‐free method (MF*: *P3* for Experiment 1–3; *P2* for Experiment 4). The horizontal dashed line represents the significance threshold with respect to the best model‐free method. In Experiment 3, *G2’* represents the US‐locked RF. For the respective winning method (*G2* for Experiment 1, 2, and 4; *G2’* for Experiment 3), Cohen's *d* was 1.33, 1.05, 1.27, and 0.59 for Experiment 1, 2, 3, 4, respectively.

We built and tested the model on the same data from Experiment 1, possibly rendering the test of the model biased. Hence, we sought to validate the model on independent datasets. The results are illustrated in Figure 4. For delay conditioning Experiment 2, model *G2* outperformed all model‐free methods, and it performed as well as models *G1* and *G3* (NLL_G2_ − NLL_G1_ = 1.74; NLL_G2_ − NLL_G3_ = 0.05, respectively). For trace conditioning Dataset 3 with a larger SOA, we first sought to determine the optimal RF. To this end, we compared two versions of *G2*: one in which the RF is time‐locked to the CS and one where it is time‐locked to the US. The US‐locked versions of *G2*, which we term *G2′*, had the highest predictive validity, thus performing better than any model‐free method, including the CS−locked version *G2*. This suggests the latency of the fear bradycardia to be time‐locked to the US. The US‐locked versions of the Models *G1–3* were not significantly different from each other (NLL_G2′_ − NLL_G1′_ = −1.13; NLL_G2′_ − NLL_G3′_ = −0.02). For Experiment 4, in which we used peripheral pulse oximetry rather than ECG to identify heart beats, *G2* outperformed all the model‐free methods and did not significantly differ from *G1* and *G3* (NLL_G2_ − NLL_G1_ = −2.66; NLL_G2_ − NLL_G3_ = 2.29). Taken together, these results demonstrated that the model G2 successfully generalizes to other, independent datasets, and guarantees a predictive validity significantly higher than classical scoring methods.

While these results imply higher sensitivity of our model‐based approach, we also sought to address its specificity. Crucially, there is no theoretical reason why the approach should overestimate CS+/CS− differences. Empirically, specificity can be assessed by investigating the difference between two CS for which the true HPR does not differ. We implemented this by randomly permuting condition labels and recomputing the HPR difference between such CS+ and CS− trials between which the true autonomic input will not be systematically different. In this analysis, we found no difference between conditions, *t*(28) = 0.95; *p* = .35, thus ensuring the unbiased nature of the method.

Our HPRF is based on 29 individuals only. To enhance generalizability of this HPRF for future studies, we sought to base it on a larger sample (i.e., combined Experiments 1, 2). This HPRF is shown in Figure 2B (bottom), and its parameters are reported in Table 2. Validation on Experiments 3, 4 showed that predictive validity for this model was not significantly different from a model based only on Dataset 1.

In our model, we assumed that fear‐conditioned bradycardia results from the convolution of an instantaneous autonomic input with our HPRF. For estimation of input amplitude in our GLM, this is mathematically equivalent to assuming any arbitrary input together with a suitable HPRF that result in the same output. Hence, we can relax this assumption and investigate possible autonomic inputs. A previous investigation has revealed early HPR to brief stimuli, for which the assumption of an instantaneous input is better justified than in the current work. Hence, we estimated the autonomic input that would result, convolved with the HPR from our previous study, in the fear‐conditioned bradycardia response observed here. Results are shown in Figure 1B and suggest a Gaussian input centered 3.8 s after the CS onset (i.e., during US delivery). Importantly, the input onset appears to occur after the CS presentations, thus ensuring the correct causality relation between stimulus and response. The finding that autonomic input may peak during the US also relates to the modeling results from Experiment 3, which suggested that the bradycardia response is time‐locked to the US, rather than the CS.

### Comparison with SCR

**Figure 5:**
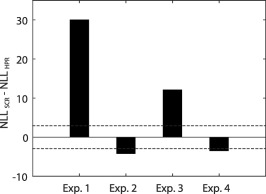
Comparison between predictive validity for HPR and SCR. The horizontal dashed lines represent the significance thresholds with respect to HPR. The HPR returns a predictive validity significantly higher than SCR for Dataset 1 and 3, while SCR is significantly better with Dataset 2 and 4.

**Figure 6:**
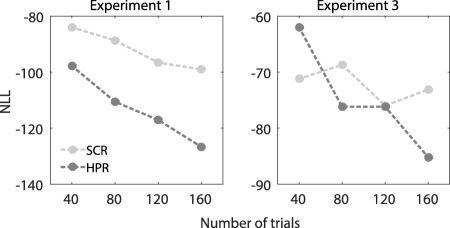
Dependence of NLL on number of trials. Modality‐specific dependence of the predictive validity as negative log‐likelihood (smaller is better) on the number of trials. The figure depicts an overall increase in predictive validity as a function of the number of trials analyzed from the beginning of the experiment, for model based approach of HPR (GLM, dark gray) and SCR (DCM, light gray).

Finally, we compared the predictive validity of the HPR estimates with SCR estimates. To do this, we contrasted the NLL obtained by the GLM implementation of the best model for the HPR (based on Dataset 1 only) with the one returned from a standard DCM analysis of the SCR on the same dataset. The results are illustrated in Figure 5, and show that the HPR allow a significantly better discrimination than SCR in Dataset 1 (NLL_HPR_ − NLL_SCR_ = −29.6) and 3 (NLL_HPR_ − NLL_SCR_ = −12.0), but not in Dataset 2 and 4, for which SCR is significantly better than HPR (Experiment 2: NLL_HPR_ − NLL_SCR_ = 4.1; Experiment 4: NLL_HPR_ − NLL_SCR_ = 3.5). We were concerned that this discrepancy might arise from the lower number of trials per condition in Experiment 2 and 4 (24 CS+US‐ in Experiment 2 and 4 in contrast with 40 CS+US‐ in Experiment 1 and 3). Hence, for the experiments with a higher number of trials (160, Experiment 1 and 3), we computed the predictive validity in dependence on the number of successive trials included into the analysis (Figure 6). If the reason for the discrepancy was the number of trials, we expected the SCR to consistently perform better when analyzing only a fraction of the dataset. Predictive validity of both HPR and SCR estimates increases with an increasing trial number. In Experiment 3, SCR outperformed HPR at low trial numbers, but in Experiment 1, HPR was always better than SCR, also for lower numbers of trials. Hence, the number of trials is probably not the reason for this discrepancy between experiments.

## Discussion

In this study, we investigated fear‐conditioned bradycardia and its suitability for quantifying human fear memory. We first showed a significant bradycardia response upon presentation of CS+ compared to CS− across three delay conditioning and one trace conditioning experiments. We then developed a PsPM that discriminates the HPR to CS+ versus CS− better than classical scoring methods. Best predictive validity was achieved with a RF that approximated the differential response as a gamma distribution that peaks 4.7 s after the CS onset, together with its time derivative accounting for between‐subjects variation in peak latency. We optimized data preprocessing and confirmed the best preprocessing settings and the best model in three independent datasets. Finally, HPR allow, on average, a better quantification of fear learning than SCR‐based estimates, although with variability between datasets. In particular, HPR performed better with a wide margin in two datasets, while it was significantly less sensitive in the remaining two. The inconsistency was not explained with the number of trials in the individual experiments. Alternative explanations may relate to the differences in the experimental designs distinguishing Experiment 2/4 from Experiment 1/3, possibly involving nonspecific effects of anticipating startling sounds or the alternation of complex and simple stimuli.

Overall, it appears that HPR is a powerful and robust indicator of fear learning, in particular when analyzed with a model‐based approach. This could be of particular importance in a neuroimaging context, since MRI machines are standardly equipped with a peripheral pulse sensor or ECG to record cardiac activity, while equipment for recording SCR is less available. However, SCR allows single‐trial estimation of fear learning (Bach, Daunizeau et al., 2010), while the single trial HPR in our data appeared to be dominated by RSA and therefore not sufficiently reliable to allow trial‐by‐trial estimation. Nevertheless, the good discriminative power of the HPR justifies future investigations aimed at developing a method capable of single trial analyses. Moreover, it would be interesting to estimate RSA independently (e.g., by integration with respiratory time series) to assess HPR uncontaminated by RSA.

An important difference between HPR and SCR is that early HPR, as analyzed in the present work, is almost exclusively modulated by the parasympathetic nervous system (Berntson et al., 2007), while SCR is under almost exclusive control of the sympathetic branch (Boucsein, 2012). Concurrently assessing both measures could provide a tool to discriminate sympathetic and parasympathetic autonomic learning.

A trace conditioning dataset was analyzed to investigate how the RF depends on CS/US interval. We found that time‐locking the RF to US performed better than locking it to the CS, in line with experimental reports showing that the second deceleration of the HPR (D_2_), that is, the component we modeled to discriminate across conditions, is time‐locked to the stimulus that is being anticipated (Damen & Brunia, 1987). This suggests that the HPR may prepare for an upcoming US, an idea in keeping with our result that an autonomic input peaking at anticipated US presentation can best explain our HPRF. However, additional datasets with more diverse SOAs are needed to unambiguously confirm this result.

To summarize, the present work provides a novel tool to evaluate fear learning. In the current state of research, where the possibility of intervening directly on memory to treat fear‐related psychiatric disorders starts being investigated, this technique provides a standardizable approach to assess fear memory. Moreover, despite its development on an ECG‐based time series, we show its validity on data from peripheral pulse oximetry, commonly available in fMRI scanners. Therefore, with its natural suitability for recordings in fMRI machines, our method may complement the current standard methods for quantifying fear memory.
